# Quality assessment of oral antimalarial and antiretroviral medicines used by public health systems in Sahel countries

**DOI:** 10.1371/journal.pone.0303289

**Published:** 2024-05-09

**Authors:** Amor R. Cáceres-Pérez, Javier Suárez-González, Ana Santoveña-Estévez, José B. Fariña

**Affiliations:** 1 Facultad de Farmacia, Universidad de La Laguna, San Cristóbal de La Laguna, Spain; 2 Programa de Doctorado Ciencias Médicas y Farmacéuticas, Desarrollo y Calidad de Vida, Universidad de La Laguna, San Cristóbal de La Laguna, Spain; 3 Instituto Universitario de Enfermedades Tropicales y Salud Pública de Canarias, Universidad de La Laguna, San Cristóbal de La Laguna, Spain; Makerere University, UGANDA

## Abstract

Malaria and Human Immunodeficiency Virus infections are among the top 10 causes of death in low income countries. Furthermore, many medicines used in these treatment areas are substandard, which contributes to the high death rate. Using a monitoring system to identify substandard and falsified medicines, the study aims to evaluate the quality of antimalarial and antiretroviral medicines in Sahel countries, assessing site conditions, compliance of medicines with pharmacopoeia tests, formulation equivalence with a reference medicine, and the influence of climate on quality attributes. Ultra Performance Liquid Chromatography methods for eight active pharmaceutical ingredients were validated following the International Conference for Harmonization guideline for its detection and quantification. Quality control consists of visual inspections to detect any misinformation or imperfections and pharmacopeial testing to determine the quality of pharmaceutical products. Medicines which complied with uniformity dosage units and dissolution tests were stored under accelerated conditions for 6 months. Artemether/Lumefantrine and Lopinavir/Ritonavir formulations failed uniformity dosage units and disintegration tests respectively, detecting a total of 28.6% substandard medicines. After 6 months stored under accelerated conditions (40 °C // 75% relative humidity) simulating climatic conditions in Sahel countries, some medicines failed pharmacopeia tests. It demonstrated the influence of these two factors in their quality attributes. This study emphasizes the need of certified quality control laboratories as well as the need for regulatory systems to maintain standards in pharmaceutical manufacturing and distribution in these countries, especially when medicines are transported to rural areas where these climatic conditions are harsher.

## 1. Introduction

Malaria and Human Immunodeficiency Virus (HIV) infections / acquired immunodeficiency syndrome (AIDS) are 2 of the top 10 causes of death in low-income countries. Furthermore, there has been a higher prevalence after the coronavirus (COVID-19) pandemic [1]. However, the mortality rate has decreased significantly, despite reports of poor quality medicines used in high prevalence areas as is the case of certain African countries [2–5]. Moreover, the resistance to first-line active pharmaceutical ingredients (API) used in the treatment of malaria and HIV has increased and thus treatment is not effective[2].

Chloroquine (CQ) is the first-line treatment of uncomplicated malaria, but in areas with high resistances to this API, lumefantrine (LMF) is considered one of the best alternatives. It is even more effective when combined with artemether (AMT) as artemisinin-based combination therapies [2,6]. Antimalarials are one of the most susceptible therapeutic groups when it comes to falsification or substandardisation. This is due, in part, to the complexity of their qualitative and quantitative analysis, as these medicines do not usually have chromophore group or have low aqueous solubility [7,8].

Regarding HIV infection, the first-line treatment consists of a combination of 2 nucleoside reverse transcriptase inhibitors (Abacavir [ABC], Lamivudine [3TC], Zidovudine [ZDV], etc.) administered with a third active antiretroviral ingredient from 1 of 3 drug classes: an integrase inhibitor (Raltegravir [RAL], Dolutegravir [DTG], Elvitegravir [EVG], etc.), a non-nucleoside reverse transcriptase inhibitor (Efavirenz [EFV], Rilpivirina [RPV], Nevirapine [NVP], etc.), or a protease inhibitor (Lopinavir [LPV], Saquinavir [SQV], Darunavir [DRV], etc.) [9]. However, there are two types of HIV, type 1 and 2, and therefore should be considered to propose a therapeutical regimen. Type 2 is more prevalent in Africa and has an intrinsic resistance to non-nucleoside reverse transcriptase inhibitors and fusion inhibitors as well as higher variability in the response against protease inhibitors [10]. Therefore, the most common APIs used against HIV-2 are a combination of 2 nucleoside reverse transcriptase inhibitors (ABC/3TC) and 2 protease inhibitors (LPV/RTV, officially the medicine is abbreviated as LPV/r) [11].

According to a World Health Organization (WHO) report, antimalarial medicines are among those medicines most frequently falsified and of substandard level. The same report indicates that antiretrovirals have the lowest rate of poor-quality detection, but they are also the least studied [12]. To harmonise procedures and results for the detection of poor quality medicines, quality test should be performed following European (Eur. Ph.) or United States Pharmacopeia (USP) recommendations. For example, in most of the published results the assay of content is completed but do not take into account the uniformity between dosage units when quality of tablets or capsules is being performed [13–16]. Another key assay is the dissolution test, which allows the quantification of dissolved APIs and consequently their potential uptake amount, providing more reliable information on their efficacy [17].

Infectious Diseases Data Observatory (IDDO) is an online platform which collects the results of the quality of medical products. It can be filtered by product category (medicines, diagnostics, etc.), by date, by country and/or therapeutic group. In the last 5 years, a total of 34 publications have been reported on the quality of antimalarial medicines and 14 publications were related to medical products used for HIV, 12 of which are directly related to the quality of medicines. Around 70% of these reports detected falsified or substandard medicine [18,19]. Other databases (PubMed, SCOPUS, etc.) do not shown many more results. Moreover, most of these studies used an inappropriate analysis methodology or did not perform all necessary tests for the same medicine [13,14,20–24]. In IDDO platform there are practically no reports in Sahel countries (Chad, Burkina Faso, Niger, Mali and Mauritania) [18,19].

In 2022, United Nations Office on Drugs and Crime reported that around 20–50% of studied medicines in Sahel countries were substandard or falsified and the 40% of them were taken from the regulated supply chain [25]. Furthermore, this issue has worsened during COVID-19 pandemic as the trafficking of medical products increased, seizing many unauthorized medicines of CQ which was one of the API that showed advantages in the treatment against this disease [25,26].

The quality of medicines may be affected at any moment during the supply chain from manufacture to dispensing. For example, India’s pharmaceutical industry was the largest exporter of generic antibiotic medicines in 2022 and led to improved global accessibility to treatment, including Sahel countries [25,27]. However, these medicines are manufactured in one country and exported to another with different climatic conditions, so it is essential to ensure the quality throughout the supply chain because medicine stability could be compromised if not properly protected from humidity and temperature changes. In this sense, the selection of the packaging is crucial as well as adhering to the International Conference Harmonisation (ICH) guideline in terms of stability [28].

The imbalance between the supply and demand of medicines, as well as poor manufacturing and distribution practices, allows the entrance of substandard and falsified medicines into the market [29]. These guidelines are mandatory, even when medicines and APIs are imported from countries outside of European Union, to ensure that they are approvable and they are also recommended for all WHO countries [30,31]. Another reason that explain why substandard medicines reach patients is the lack of WHO certified and accredited laboratories which carry out the quality control of medical products at different points of the supply chain [29].

Currently, The African Union is working to stablish an African Medicine Agency (AMA) whose objectives are to coordinate national and subregional medicines regulatory systems, carry out regulatory oversight of selected medical products, promote cooperation, ensure access to affordable medicines and the harmonization and mutual recognition of regulatory decisions.

Thus, initiatives such as ISACAM (MAC2/1.1a/219), a European project that aims to ensure the quality of medicines used in the treatment of HIV, malaria and tuberculosis in Mauritania, have emerged. This project has been carried out in collaboration with Laboratoire National de Contrôle de la Qualité des Médicaments (LNCQM, from French), and currently applying to achieve the WHO certification on quality control of medicines. In addition, the first report on the quality of anti-tuberculosis medicines in Mauritania has recently been published [32].

The aim of the current work was to assess the quality of antimalarial and antiretroviral medicines used in Mauritania, as a representative country with quality control laboratory, employing a monitoring system to determine the quality of these medicines and possible flaws. The complete monitoring of the quality of the medicines included the evaluation of the conditions at the sampling sites, the determination of compliance of pharmacopeial test specifications, the comparison of the dissolution profiles between formulations to establish their equivalence or not, and the study of climatic conditions influence on their quality attributes.

## 2. Materials and methods

### 2.1. Materials

The APIs used for this study were: CQ (Sigma-Aldrich^®^, United State of America [USA]), AMT and LMF (USP reference standard, USA), ABC, 3TC, ZDV, RTV and LPV (European pharmacopeia reference, France). Other required products for the tests were formic acid, ammonium acetate (Merck^®^, Germany), chloride acid (Fluka^™^, Germany), acetonitrile, methanol (Honeywell^®^, Germany), potassium dihydrogen phosphate, tetrahydrofuran (THF), polyoxyethylene (10) lauryl ether and benzalkonium chloride (Sigma-Aldrich^®^, Germany). Pure water was obtained through Smart2Pure Pro UV/UF 16 LPH system (Thermo Scientific^®^, USA).

### 2.2. Sampling

The selection and sampling of medicines was done considering efficacy to treat the diseases, availability in Mauritania, and prioritizing the care of the patients in order to avoid any possible damage to the Mauritanian Health System. Samples were taken in the Spanish and Mauritanian National Health Systems. An adequate number of dosage units (n ≥ 120) were sampled from the same batch of each medicine in order to be able to perform all the tests foreseen. Antimalarial medicines were sampled from distribution companies and the Programme National de Lutte contre Tuberculosis et Paludisme (PNLTP, from French), where samples are directly obtained from suppliers. Antiretroviral medicines were taken from distribution companies and pharmacies. During sampling, a datasheet was completed *in situ* and the information related to date, packaging (condition, information contained, possible misinformation etc), the storage place (type of establishment, temperature, humidity etc) and the person responsible of the sampling was noted. Then, formulations were transported in isothermal bags to the laboratory to perform a quality control. In the lab, they were stored at 5 °C and 11% of relative humidity (RH) and analysed before their expiry dates whenever possible.

### 2.3. Analytical methods validation

The analysis of APIs was carried out using an Acquity Ultra Performance Liquid Chromatography (UPLC)^®^ H-Class System (Waters Corporation^®^, USA) and the data was acquired by Empower 3 software (Waters Corporation^®^, USA). All methods were adapted from High Performance Liquid Chromatography methods available in USP for each medicine [33–36].

Each analytical method was validated following the ICHQ2(R1) guideline [37], employing Excel^®^ software (Microsoft Corporation^®^, USA) for data processing and to analyse the origins of variation. The linearity was confirmed through the rejection of a null hypothesis (α = 0.05), the accuracy (expressed as recovery percentage, 97–103%) was determined by analysis of a known concentration of standard (n = 9), precision (expressed as repeatability, <1% for UV and <5% for mass detection) was calculated by testing the same sample six times. The limit of detection (LOD) and quantification (LOQ) were also determined.

All standard solution and samples were filtered by 0.2 μm pore-size filter (Macherey-Nagel^®^, Germany) before their analyses. Every working day a standard solution was used to ensure the proper performance of the instrument.

#### 2.3.1. Antimalarial APIs

For antimalarial APIs: two different isocratic analytical methods were adapted using a XSelect CSH C18 column (75 x 2.1 mm, 2.5 μm) as stationary phase. It was maintained at 25 °C for CQ and 30 °C for the combination of AMT/LMF. The flow rate and injection volume were 0.4 ml/min and 10 μl.

The mobile phase of CQ method was Methanol: Phosphate Buffer (22:78, v/v). In order to fulfil USP recommendations two wavelengths were used, 224 and 343 nm for uniformity of dosage unites and dissolution test respectively. A standard solution of 0.14 mg/ml was prepared and diluted to a concentration range of 2.8–14 μg/ml with mobile phase for method validation.

The mobile phase used for AMT/LMF method was Methanol: 0.1% Formic Acid in water (15:85, v/v). The detection of LMF was done at 236 and 342 nm uniformity of dosage units and dissolution test, respectively. In addition, an Acquity Triple Quadrupole (QDa, Waters^®^, USA) was coupled to UPLC to detect and quantify AMT because it has no chromophore group [38]. In this case, a selected ion recording (SIR) of 163 m/z was used for the detection and quantification of this API. The conditions employed for mass detection were: positive mode electrospray ionization, 600 °C as source and desolvation temperature and a capillary and cone voltage of 0.8 kV and 30 V. Nitrogen was used as a desolvation gas. For the preparation of the standard solution of AMT/LMF, a mixture of the same proportion of APIs as is present in medicine (1:6) was dissolved in 8 ml of THF and sonicated for 15 minutes. The volume was filled up to 10 ml with pure water and concentrations of 0.2 and 1.2 mg/ml were obtained for AMT/LMF. Finally, it was diluted to obtain concentrations from 0.2 to 24 μg/ml for both APIs using mobile phase as diluent (S1 Table).

#### 2.3.2. Antiretroviral APIs

In the case of LPV and RTV, one isocratic method was adapted using an Acquity Premier BEH C18 column (2.1 x 100 mm, 1.7 μm) as a stationary phase at a temperature of 25 ºC. A flow rate of 0.4 ml/min of a mixture of Acetonitrile and Potassium Phosphate Monobasic Buffer (55:45, v/v), adjusted to a pH of 4.0 with phosphoric acid, was used as mobile phase. For these APIs a wavelength of 215 nm was used. Standard solutions with concentrations of 2.0 and 0.5 mg/ml were prepared in methanol, for LPV and RTV respectively, and diluted with mobile phase to a concentration range between 2.5 and 25 μg/ml.

Quantification of the rest of antiretroviral APIs studied (ABC, ZDV and 3TC) was carried out using an Ammonium Acetate aqueous solution (1.9 g/L) adjusted to a pH of 3.9 with Acetic Acid (solution A) and Methanol (solution B) in gradient. The initial conditions of the gradient elution were 97% of solution A with a flow rate of 0.4 mL/min. The proportion of solution A is changed to 76% at 1.00 minutes and to 40% at 3.50 minutes. Then, it was kept in that conditions for 1.50 minutes and subsequently initial conditions were stablished for re-equilibration. The column was set up to 30 °C, the injection volume was 10 μl and UV detection was done at 270 nm. A mass of each API was weighted, dissolved in solution A and sonicated for 5 minutes until obtaining solutions of 3 mg/ml for 3TC and 0.6 mg/ml for ZDV and ABC. Next, they were diluted with solution A from 1.5 and 3 to 9 and 18 μg/ml, respectively (S2 Table).

### 2.4. Quality control assays

The quality control of oral dosage forms included a visual inspection and the tests recommended by Eur. Ph. and USP for all drug products studied [39,40].

#### 2.4.1 Visual inspection

Visual inspection was carried out at the time of medicine sampling, checking that they contained the regulatory information necessary (API name, dose and dosage form as well as the manufacture and expiry date, batch number, number of units and the name of manufacturer laboratory). In addition, an examination was carried out for imperfections, colour changes or any other relevant aspect during storage under accelerated conditions (40 °C/ 75% RH) [28]. Furthermore, a digital caliper (Sesa Tools^®^, Spain) was used to determine the size of 3 tablets.

#### 2.4.2 Mass uniformity test

This test was performed according to Eur. Ph. by weighing 20 random tablets, calculating the average and standard deviation of the tablets and checking that the individual weights do not deviate more than indicated [41].

#### 2.4.3 Uniformity of dosage units test

Mass variation or content uniformity tests were carried out according to the Eur. Ph. calculating the acceptance value (AV) to ensure the uniformity between units, being 15 as the pharmacopeial limits [42]. All samples were prepared and diluted following the standard solution procedure (see Validation of analytical methods section).

#### 2.4.4 Dissolution test

Dissolution tests were performed following USP recommendations, see Table 1 [43]. In the case of AMT/LMF tablets, as an official monography is not available, different mediums were studied based on literature [34,44]. For all medicines, paddle apparatus was used [33,34,36,45]. DT 1410 dissolution tester (Erweka^®^, Germany) was used and samples were filtered (3 mm 46 x 57 cm Whatman^®^, United Kingdom), diluted and analysed by UPLC-UV or UPLC-MS.

**Table 1:** Dissolution test conditions for antimalarials and antiretrovirals sampled medicines.

API	Medium and volume	Agitation (time and rpm)	Q (%)
**CQ**	Water, 900 ml	45 minutes; 100 rpm	75
**AMT**	1% of benzalkonium chloride in HCl 0.1 N, 1000 ml	45 minutes; 100 rpm	40
**LMF**			60
**ABC**	HCl 0.1 N, 900 ml	30 minutes; 75 rpm	80
**LMV**			
**LPV**	60 mM polyoxyethylene 10 lauryl ether in water; 900 ml	90 minutes; 75 rpm	80
**RTV**			

API: Active pharmaceutical ingredient. CQ: Chloroquine phosphate. AMT: Artemether. LMF: Lumefantrine. ABC: Abacavir sulphate. 3TC: Lamivudine. ZDV: Zidovudine. LPV: Lopinavir. RTV: Ritonavir. HCl: Chloride Acid. Q: Tolerance value.

When an European equivalent formulation was available, a dissolution profile comparison between Mauritanian and European medicines was performed through the value of *f2* (Eq 1), following the European Medicine Agency (EMA) recommendations [46]. In this case, the European formulation was set as reference medicine and Mauritanian was identified as the test formulation.

$$f2=50*\;\text{log}(\sqrt{1+\frac{1}{n}\cdot \sum_{t=1}^{n}{\left(R_{t}-T_{t}\right)}^{2}}\cdot 100)$$ (1)

Where Rt and Tt are the released percentage at each sampling time for reference (Resochín^®^) and test medicines (CQ tablets) respectively. n is the number of points used.

#### 2.4.5 Disintegration test

Disintegration time was determined using a DIST-3 disintegrator tester (Pharma-test^®^, Germany) according to Eur. Ph. recommendations for uncoated and coated tablets [47].

#### 2.4.6 Friability test

As stated in the Eur. Ph. monography, this test was only carried out for uncoated tablets in a Tablet Friability/Abrasion Tester TAR Series (Erweka^®^, Germany) [48].

### 2.5. Stability studies of medicines

Medicines which complied with uniformity of dosage units and dissolution requirements were stored under accelerated conditions (40 °C and 75% RH) in an ICH 110L climatic chamber (Memmert^®^, Germany). Then, samples were analysed after 3 and 6 months of storage to verify the compliance of specifications.

## 3. Results and discussion

### 3.1. Sampling

A total of 7 medicines were sampled from Mauritania and Spain, 4 were antimalarial pharmaceutical products and the other 3 were formulations to treat HIV. In Table 2, the APIs, dose and pharmaceutical dosage form of medicines sampled, the manufacturer country, the batch number and the sampling place are shown.

**Table 2:** List of samples.

Brand name	API and dose (mg)	Pharmaceutical dosage form	Manufacturer Country	Batch number	Sampling place
**Chloroquine phosphate tablets**	CQ 250	Film coated tablets	Cyprus	82793	Mauritania (Nouakchott)—PNLTP
**Resochín** ^**®**^			Spain	R001	Spain (Tenerife)–Distribution company
**Lumiter** ^**®**^	AMT 20 LMF 120	Uncoated tablets	India	HP/L/Drugs/12/729	Mauritania (Nouakchott)–PNLTP
**Artemether and Lumefantrine tablets**			India	HWE199027	Mauritania (Nouakchott)—PNLTP
**Abacavir and Lamivudine**	ABC 600 3TC 300	Film coated tablets	Spain	BD6020012A	Spain (Tenerife)–Distribution company
**d’abacavir et lamivudine comprimés USP**			India	ABL21019	Mauritania (Nouakchott)–Pharmacy
**Lopinavir and Ritonavir**	LPV 200 RTV 50		India	3126547	Mauritania (Nouakchott)–Pharmacy

USP: United State Pharmacopeia. API: Active Pharmaceutical Ingredients. PNLTP: Programme National de Lutte contre Tuberculosis et Paludisme. CQ: Chloroquine phosphate. AMT: Artemether. LMF: Lumefantrine. ABC: Abacavir sulphate. 3TC: Lamivudine. ZDV: Zidovudine. LPV: Lopinavir. RTV: Ritonavir.

CQ was included in this work because a study conducted in Mauritania demonstrated 100% efficacy in the treatment of acute malaria, despite of CQ is not widely used in Africa due to the resistance in endemic areas [49]. There are some reports on the quality of antiretroviral medicines concluding that they met the requirements although there is no register of their manufacturer or batch number [50,51]. However, these medicines should not be available on the market because of their questionable traceability.

### 3.2. Analytical methods validation

**Fig 1:**
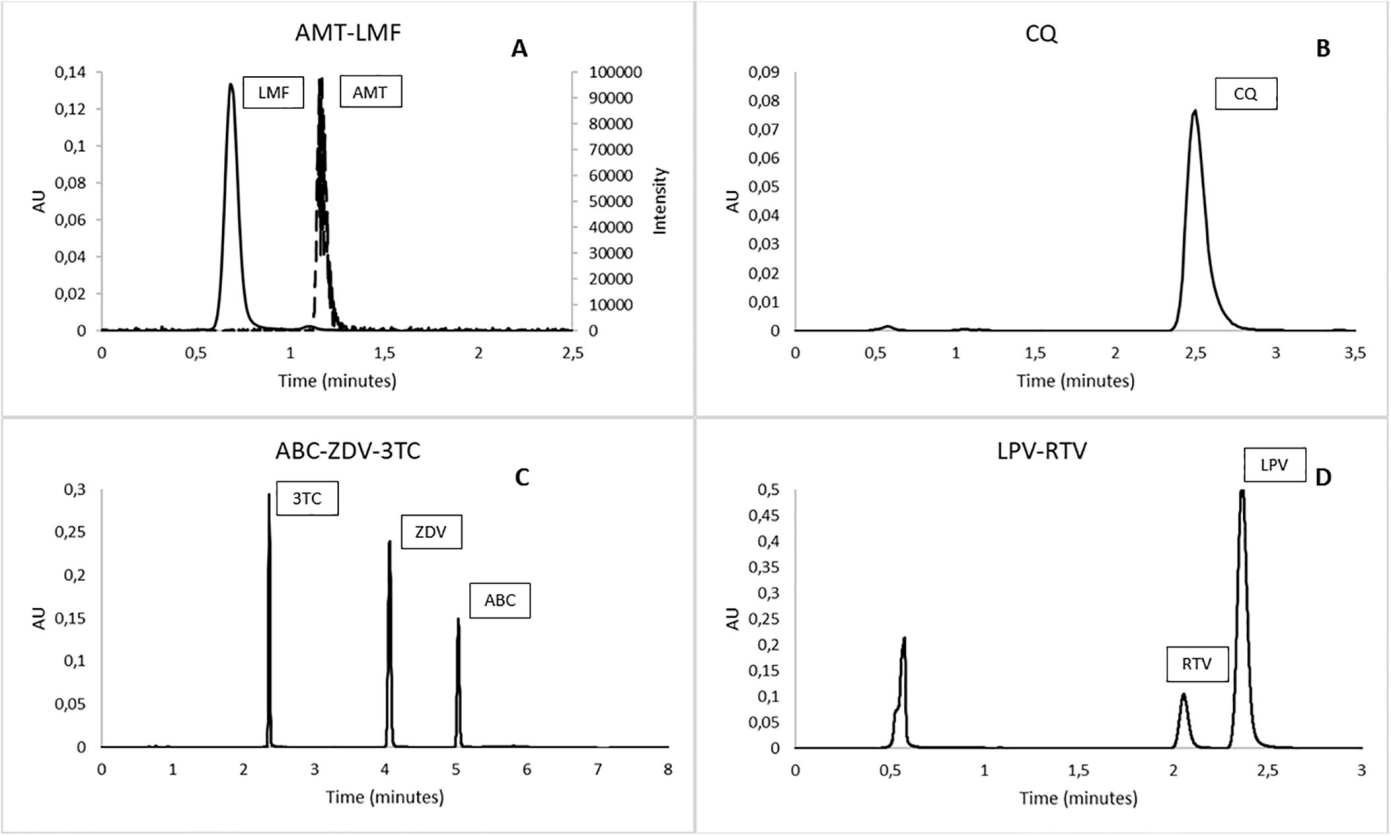
Chromatograms of each analytical method. A: Lumefantrine (Rt: 0.665 min) and Artemether (Rt: 1.126 min). B: Chloroquine phosphate (Rt: 2.511 min). C: Lamivudine (Rt: 2.237 min), Zidovudine (Rt: 4.003 min) and Abacavir sulphate (Rt: 5.006 min). D: Mobile phase (Rt:0.524 min), Ritonavir (Rt: 2.110 min) and Lopinavir (Rt: 2.378 min). Rt: Retention time.

A total of 4 UPLC methods were validated to analyse 8 APIs. The chromatographic resolution between peaks was adequate as is shown in Fig 1. Continuous line indicated UV chromatogram and discontinuous line is for SIR channel.

**Fig 2:**
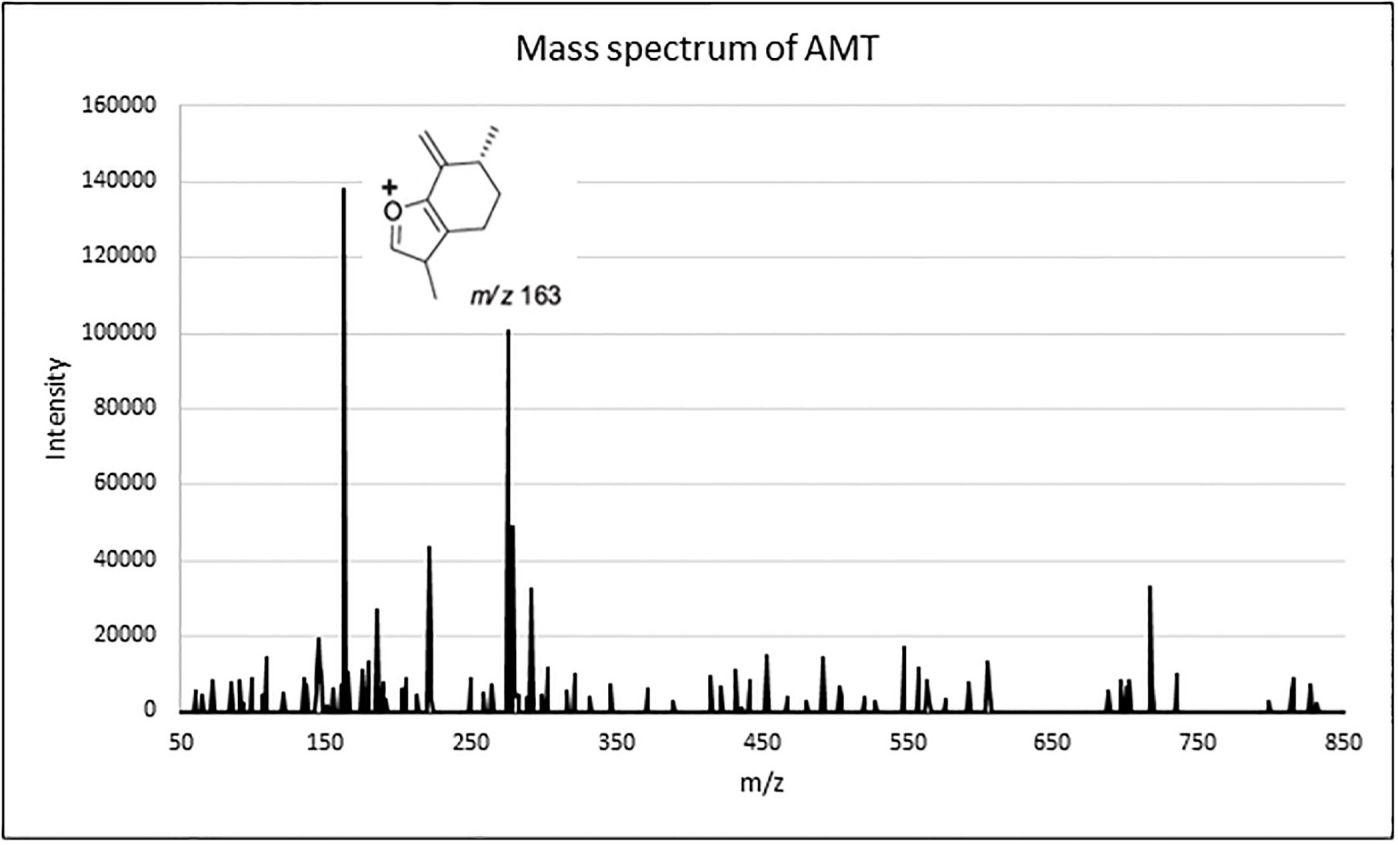
Mass spectrum of artemether. Chemical structure related to 163 m/z is shown.

The detection and quantification of AMT was carried out at 163 m/z because it was the main signal obtained in the mass spectrum under chromatography conditions as show in Fig 2. In addition, this m/z signal was used in previous methods and the fragmentation of AMT was described in literature [52,53].

The UPLC-MS is a more selective and specific technique than UPLC-UV because it allows to differentiate APIs, impurities and degradation products through the m/z signal obtained in the scan mode. However, the first technique is also more complex because there are more origins of variations that influence in the obtained signal but provide a good coefficient of variation (CV) in comparison with other validated methods used for the same purpose [54]. The results of analytical method variation of each API are shown in Table 3.

**Table 3:** Results of antimalarial and antiretroviral analytical methods.

API		Equation	CV (%)	Precision (%)	Accuracy (%)	LOD (μg/ml)	LOQ (μg/ml)
**CQ**	**224 nm** *	A = 75638.20·C (R^2^ = 0.992)	3.66	0.10	98.5	0.9	2.7
	**343 nm** *	A = 49916.93·C (R^2^ = 0.996)	2.69	0.11	98.7	0.7	2.9
**AMT**		A = 42373.07 + 161078.67·C (R^2^ = 0.992)	7.30	3.90	102.3	0.4	1.3
**LMF**	**236 nm** *	A = 114248.59·C (R^2^ = 0.999)	3.75	0.88	101.5	1.3	4.0
	**342 nm** *	A = 45111.50·C (R^2^ = 0.995)	4.34	2.23	101.8	0.7	2.0
**ABC**		A = 38349.87·C (R^2^ = 0.990)	4.50	0.46	100.9	1.6	4.7
**3TC**		A = 83109.89·C (R^2^ = 0.994)	3.75	0.64	98.2	0.7	2.0
**ZVD**		A = 68765.14·C (R^2^ = 0.993)	3.78	0.06	101.9	1.3	4.0
**LPV**		A = 49242.68·C (R^2^ = 0.998)	2.65	0.40	100.0	1.2	3.7
**RTV**		A = 40569.14·C (R^2^ = 0.983)	4.80	0.54	98.7	1.0	3.0

API: Active pharmaceutical ingredients. CQ: Chloroquine phosphate. AMT: Artemether. LMF: Lumenfantrine. ABC: Abacavir sulphate. 3TC: Lamivudine. ZDV: Zidovudine. LPV: Lopinavir. RTV: Ritonavir. A: Area (μV/s). C: Concentration (μg/ml). CV: Coefficient of variation. LOD: Limit of detection. LOQ: Limit of quantification.

* Indicates the wavelength in which the API was measured.

For a better understanding, the results of the quality control and stability study performed for each therapeutic group of sampled medicinal products are shown together.

### 3.3 Antimalarial medicines

#### 3.3.1 Quality control

**Fig 3:**
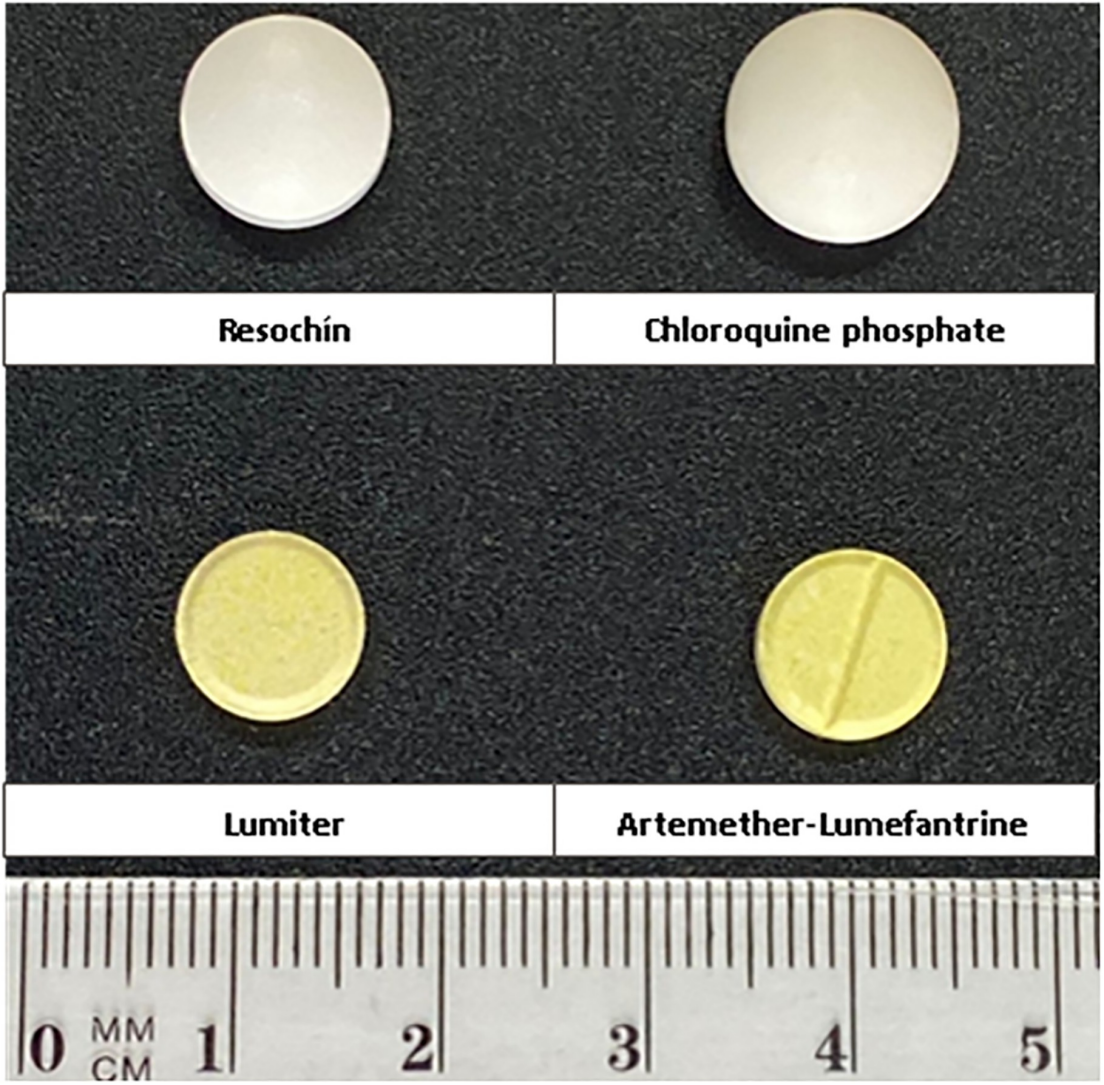
Appearance of each antimalarial medicine. Size is expressed as mean ± standard deviation (n = 3) for Resochín^*®*^ (10.18 ± 0.03 and 5.42 ± 0.02 mm), chloroquine phosphate tablets (11.54 ± 0.02 and 6.37 ± 0.02 mm), Lumiter^*®*^ (9.06 ± 0.02 and 3.59 ± 0.01 mm) and Artemether and lumefantrine tablets (9.17 ± 0.02 and 3.18 ± 0.02 mm), First parameter is diameter and second is height.

Imperfections or misinformation were not detected in primary and secondary packaging. In Fig 3, the appearance of each antimalarial medicine is shown.

The results of pharmacopeial test of antimalarial medicines are shown in Table 4.

**Table 4:** Results of pharmacopeial test for antimalarial medicines.

Brand name	API	Mass Uniformity (g), n = 20 [min–max]	Disintegration time, n = 6	Dissolution (% released), n = 6	UDU (% content) [AV]	Friability (%)
**Resochín** ^**®**^	CQ	0.427 ± 0.004 [0.421–0.437]	13’ 08”	93.3 ± 3.0	97.2 ± 1.0 [3.8]	NA
**Chloroquine phosphate**	CQ	0.538 ± 0.011 [0.511–0.565]	4’ 55”	88.5 ± 1.6	97.2 ± 1.9 [5.9]	NA
**Lumiter** ^**®**^	AMT	0.251 ± 0.003 [0.245–0.257]	2’ 55”	94.3 ± 5.2	101.1 ± 1.6 [10.2]	0.14
	LMF			98.8 ± 10.7	101.6 ± 4.2 [3.8]	
**Artemether-Lumefantrine**	AMT	0.252 ± 0.002 [0.249–0.252]	3’ 38”	56.0 ± 3.1	104.0 ± 5.4 [13.3]	0.12
	LMF			83.1 ± 4.8	106.4 ± 5.2 [15.4]	

API: Active pharmaceutical ingredient. CQ: Chloroquine phosphate. AMT: Artemether. LMF: Lumefantrine. UDU: Uniformity of Dosage Units. AV: Acceptance value. NA: No applicable. Shadow cells indicated substandard medicines and the cause.

CQ formulations fulfil all pharmacopoeial recommended tests, being standard medicines. In previous studies done in Sahel countries, substandard CQ medicines were detected by dissolution test failures or lower and greater API content detection than the labelled dose [20,21]. However, it was not possible to define the cause of these failures as the technical data sheet was not available to study the API polymorphism changes under accelerated conditions due to the potential API-excipients interactions [55].

In that sense, AMT/LMF generic tablets did not meet uniformity of dosage units test because the AV was greater than 15, and units with around 116% API content were detected, which could be harmful due to overdosing. Based on pharmacopeial recommendations, the performed test was content uniformity to evaluate the homogeneity of dosage units due to factors as the potential segregation during manufacturing [56], even despite the advances on analytical tools to perform the mass variation test on these cases [57].

Regarding the dissolution test, the water medium was tested but LMF did not dissolve, even after 3 hours, because it is insoluble in water and AMT was partly dissolved as previous reports indicated [44]. Then, 0.1 N chloride acid (HCl) containing 1% benzalkonium chloride was used to perform the AMT/LMF tablets dissolution test just employing 6 units and not 12 (in the first stage of dissolution test) as the proposed USP monograph recommends [34]. As may be seen in Table 4 both formulations complied with this test, as more than 60% and 40% of LMF and AMT was dissolved in less than 45 minutes. Although, medicines complied with dissolution test, the 40% of difference in the AMT release during the test for Lumiter^®^ should be highlighted in comparison with AMT/LMF generic tablets because it may be consequences in the treatment efficacy.

**Fig 4:**
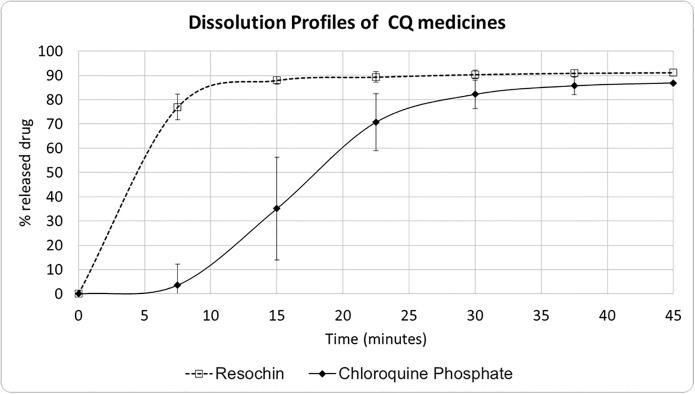
Dissolution profile of chloroquine phosphate formulation. CQ: Chloroquine phosphate.

Fig 4 shows the dissolution profiles of CQ formulations (S3 and S4 Tables). In this assay the difference between dissolution rate is also noticeable as more than 85% of CQ from Resochín^®^ dissolves in 15 minutes while the CQ generic formulation did not reach this percentage. Taking into account these results there is not enough sampling time points to apply *f2* statistics according to EMA recommendations (Eq 1). The dissolution profile of Mauritanian medicine can be affected by the country’s climatic conditions as Risha et al. demonstrated after the storage of the same batch of one CQ medicine under accelerated conditions [58].

#### 3.3.2 Stability study

All antimalarial medicines complied with tests after being stored under accelerated conditions as shown in Table 5. However, at 3 months the packaging had already opened, and therefore the exposure of the dosage forms to humidity and temperature was more relevant compared to the dissolution test at 6 months, as the packaging was sealed. In addition, the phosphate salt of CQ is an hygroscopic substance and the % of RH may affect the dissolution properties, as Lalíc-Popovíc et al. demonstrated [59,60]. This salt also has two polymorphs, and it has consequences on the API solubility [61,62]. Based on these results, the humidity appears to have several consequences on the dissolution behaviour of CQ.

**Table 5:** Antimalarial medicine stability results.

		t = 3 months					t = 6 months				
		UDU test			Dissolution test		UDU test			Dissolution test	
Brand Name	API	% DV	AV	n	% released	n	% DV	AV	n	% released	n
**Resochin** ^**®**^	CQ	100.6 ± 1.0	2.5	10	86.7 ± 3.3	6	101.2 ± 1.2	2.9	10	103.6 ± 2.3	6
**Chloroquine phosphate**		103.0 ± 2.1	6.6	10	82.9 ± 4.3	12	103.6 ± 1.3	5.1	10	96.9 ± 2.3	6
**Lumiter** ^**®**^	AMT	107.5 ± 2.4	11.8	10	97.1 ± 5.7	6	101.7 ± 5.5	13.3	10	95.7 ± 6.4	6
	LMF	106.4 ± 3.1	14.4		95.3 ± 9.7		105.8 ± 3.7	13.3		81.3 ± 5.2	

API: Active pharmaceutical ingredient. CQ: Chloroquine phosphate. AMT: Artemether. LMF: Lumefantrine. UDU: Uniformity of dosage units. DV: Declared value. AV: Acceptance value. n: number of samples.

### 3.4 Antiretroviral medicines

#### 3.4.1 Quality control

**Fig 5:**
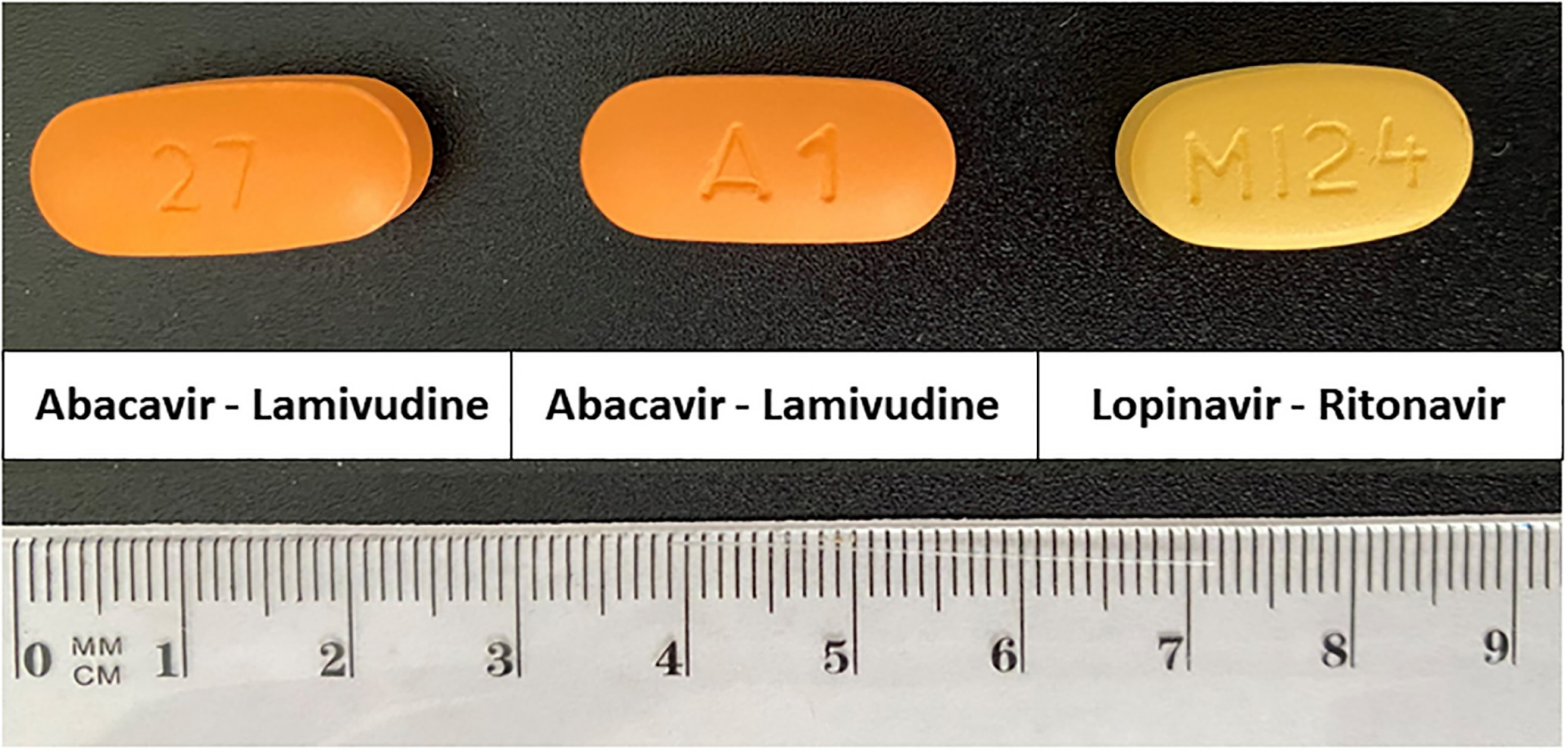
Appearance of antiretroviral tablets. Size is expressed as mean ± standard deviation (n = 3). From left to right: Abacavir-Lamivudine/Spain (21.16 ± 0.07; 9.36 ± 0.02 and 8.54 ± 0.01 mm), Abacavir-Lamivudine/Mauritania (20.90 ± 0.02; 9.25 ± 0.01 and 8.34 ± 0.02 mm) and Lopinavir-Ritonavir/Mauritania (18.94 ± 0.01; 10.13 ± 0.01 and 8.11 ± 0.03 mm), First parameter is the large, next to the width and the last one is the height.

The visual inspection of the antiretrovirals was carried out without detecting imperfections and lack of information on the packaging. The LPV/r medicine has a moisture trapping device inside the bottle to avoid any moisture after opening. The appearance of each formulation is shown in Fig 5.

The results of pharmacopeial tests which were carried out for antiretrovirals quality control are shown in Table 6.

**Table 6:** Results of pharmacopeial test for antiretroviral medicines.

Branded Name	API	Mass Uniformity (g), n = 20 [min–max]	Disintegration time, n = 6	Dissolution (% released), n = 6	UDU (% content) [AV]
**Abacavir and Lamivudine**	ABC	1.420 ± 0.006 [1.409–1.436]	6’ 20”	97.4 ± 1.7	104.7 ± 2.6 [9.4]
	3TC			111.0 ± 2.0	99.1 ± 1.8 [4.3]
**d’abacavir et lamivudine comprimés USP**	ABC	1.389 ± 0.006 [1.380–1.399]	6’ 36”	99.6 ± 1.4	104.0 ± 2.9 [9.6]
	3TC			97.5 ± 1.8	103.8 ± 3.6 [10.8]
**Lopinavir & Ritonavir**	LPV	1.247 ± 0.012 [1.224–1.270]	> 30’	100.7 ± 1.4	103.3 ± 2.8 [8.7]
	RTV			92.2 ± 2.2	102.0 ± 3.3 [8.4]

API: Active pharmaceutical ingredient. ABC: Abacavir sulphate. 3TC: Lamivudine. ZDV: Zidovudine. LPV: Lopinavir. RTV: Ritonavir. AV: Acceptance value. Shadow cells indicated substandard medicines and the cause.

The mass uniformity test was satisfactory for all formulations. The mean weight of each medicine was greater than 250 mg and no unit deviated more than the pharmacopeial criteria (5%). A previous report indicated that most of the sample quality assurance studies complied specifications regarding this test [63].

According to their mean mass and the declared value of each API, the test performed for uniformity of dosage units was content uniformity. All formulations complied with the test because the obtained AV for each API and medicine studied was less than 15%. However, some published studies just performed assay test taking into account the % content of the sample and the possible impurities without considering the homogeneity between dosage units [13,14].

**Fig 6:**
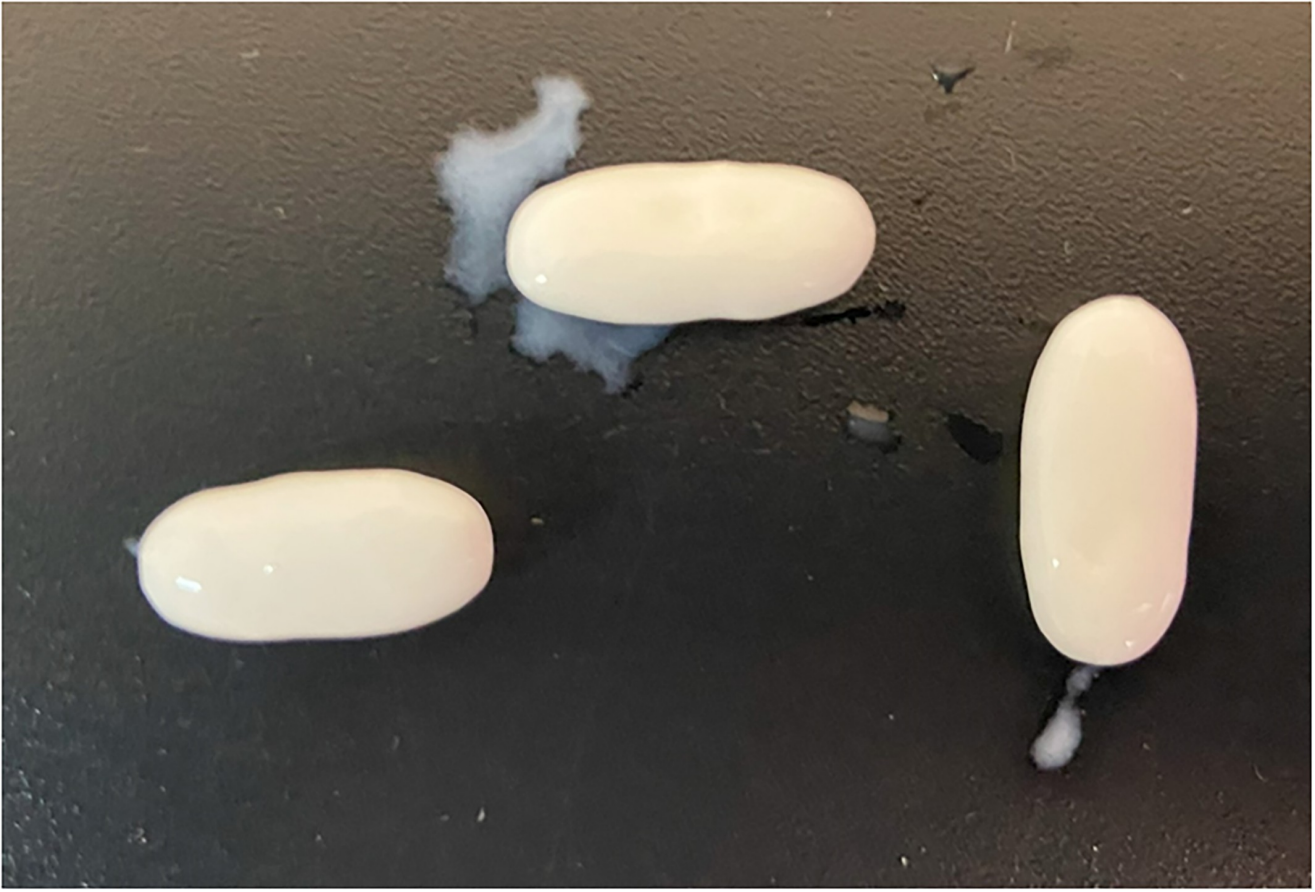
LPV/r tablets after 30 minutes in 0.1N chloride acid.

The disintegration time of each formulation is indicated in Table 6. LPV/r tablets did not comply this test because the tablets did not disintegrate in water in less than 15 minutes. As Eur. Ph. recommends, the medium was changed to HCl 0.1 N but the dosage unit remained unchanged after 30 minutes in this medium, as can be observed in Fig 6. In 2021, Sophia et al. published a case study on the quality of antiretrovirals in Tanzania in which 7 substandard LPV/r medicines were detected due to failure of the disintegration test [14]. However, as included in a review done by Do Ngan et al. [50], there were also substandard LPV/r medicines because they did not comply with uniformity of dosage units test even when the number of dosage units analysed is lower than 10 units [24]. Furthermore, both APIs are classified as IV class in the Biopharmaceutical Classification System, and RTV has a precipitation behaviour when it changes from acidic to other more basic mediums. So, it affects the bioavailability because they would not be susceptible to absorption [64,65].

All medicines complied dissolution test for 6 tablets, even LPV/r tablets which kept part of their structures after 1.5 hours of testing and the APIs are poorly water soluble [66,67].

#### 3.4.2 Stability study

Although LPV/r tablets were substandard at initial time, this formulation was stored to assess the influence of accelerated conditions on content and dissolution quality attributes. After storage under accelerated conditions, medicines were analysed again at 3 and 6 months and no changes in packaging or dosage units were detected, see Table 7.

**Table 7:** Stability study results for antiretroviral medicines.

		t = 3 months					t = 6 months				
		UDU test			Dissolution test		UDU test			Dissolution test	
Branded Name	API	% DV	AV	n	% released	n	% DV	AV	n	% released	n
**Abacavir and Lamivudine**	ABC	94.9 ± 4.5	14.3	10	101.1 ± 1.7	6	93.5 ± 2.1	10.1	10	89.7 ± 1.3	12
	3TC	99.7 ± 5.1	12.2		97.4 ± 2.6		93.4 ± 2.0	9.9		85.6 ± 1.5	
**d’abacavir et lamivudine comprimés USP**	ABC	104.0 ± 2.9	9.6	10	99.6 ± 1.4	6	95.1 ± 5.3	16.1	10	69.5 ± 2.7	12
	3TC	103.8 ± 3.6	10.8		97.5 ± 1.8		96.3 ± 2.8	9.1		66.8 ± 2.2	
**Lopinavir & Ritonavir**	LPV	101.7 ± 3.9	9.6	10	96.5 ± 1.5	6	103.4 ± 3.1	9.4	10	96.1 ± 4.5	6
	RTV	94.5 ± 3.3	11.9		92.4 ± 3.0		101.9 ± 2.3	5.9		94.4 ± 4.3	

API: Active pharmaceutical ingredient. ABC: Abacavir sulphate. 3TC: Lamivudine. ZDV: Zidovudine. LPV: Lopinavir. RTV: Ritonavir. UDU: Uniformity of dosage units. DV: Declared value. AV: Acceptance value. n: number of samples. Shadow cells indicated substandard medicines and the cause.

All formulations continued to comply with the uniformity dosage units and dissolution tests at 3 months but not at 6 months of storage. ABC and 3TC Mauritanian medicine did not comply with dissolution test for 12 units because 4 units released less than 65% (Q– 15%). This formulation also failed the uniformity of dosage units test because of the high variation between units for ABC content, even 4 units were below than 90%. Medicine expired while it was stored, but this result may explain the cause of any substandard medicine of ABC/3TC due to storage conditions.

## 4. Conclusion

This work offers faster and reliable analytical methods for performing quality control tests which were adapted from USP. Furthermore, in the case of AMT/LMF tablets, a method that allows the analysis of medicines is provided that follows international recommendations, although a mass detector is required to quantify the AMT.

Despite of the low availability and the supremacy of caring for people over performing quality control of medicines, that can be sampled and analysed, 28.6% of all medicines tested were categorised as substandard. One was an antimalarial medicine (AMT/LMF generic formulation), and the other was an antiretroviral medicine (LPV/r), both from the Mauritanian Health System and manufactured in India. The first medicines did not comply with uniformity of dosage units test and thus the treatment could be ineffective because the labelled dose of each unit is not ensured. The antiretroviral did not disintegrate, leading to consequences regarding the absorption, bioavailability and therapeutic regimen of the formulation. Medicines which were used for the stability study, continued to be standard, except for ABC/3TC USP tablets that did not comply with specifications at 6 months probably due to the storage conditions.

In the future, sampling sites should be extended to rural areas where, given the long distances and transport times involved, the quality of medicines may be affected. In addition, the economic situation in these areas is deficient compared to urban areas and this may lead to a lack of adequate infrastructure for storage and dispensing of these medicines.

The achievement of certification of quality control laboratories and the establishment of the regulatory agency are essential to ensure the quality of the medicines that are commercialized in the public Health System of the Sahel countries and work against the illegal trafficking of medicines.

## Supporting information

S1 Table Summary of analytical method conditions for antimalarials. API: Active Pharmaceutical Ingredient. CQ: Chloroquine phosphate. AMT: Artemether. LMF: Lumefantrine. T: Temperature. Q: Flow. Inj. Vol.: Injection volume.
(DOCX)

S2 Table Antiretroviral analytical method conditions. API: Active pharmaceutical ingredient. LPV: Lopinavir. RTV: Ritonavir. ABC: Abacavir sulphate. 3TC: Lamivudine. ZDV: Zidovudine. T: Temperature. Q: Flow. Inj. Vol.: Injection volume. *It is specified in the text.
(DOCX)

S3 Table Dissolution profile data of generic chloroquine phosphate medicine. Data provided per unit (n) is expressed as CQ % released at each time.
(DOCX)

S4 Table Dissolution profile data of Resochín^*®*^ medicine. Data provided per unit (n) is expressed as CQ % released at each time.
(DOCX)
