# Supplementary material for: Quality assessment of oral antimalarial and antiretroviral medicines used by public health systems in Sahel countries
Source: PLoS One. 2024 May 9;19(5):e0303289. doi: 10.1371/journal.pone.0303289 (PMC11081281; doi:10.1371/journal.pone.0303289)
Supplement: S1 Table — API: Active Pharmaceutical Ingredient. CQ: Chloroquine phosphate. AMT: Artemether. LMF: Lumefantrine. T: Temperature. Q: Flow. Inj. Vol.: Injection volume. (DOCX) [file pone.0303289.s001.docx]

**Supporting Information**

**S1 Table. Summary of analytical method conditions for antimalarials.**

| **API** | **Column** | **T (ºC)** | **Q (ml/min)** | **Inj. Vol**  **(µL)** | **Mobile phase** | **Detection** | **Concentration Range (µg/mL)** |
| --- | --- | --- | --- | --- | --- | --- | --- |
| **CQ** | XSelect CSH C18 [75x2.1 mm, 2.5 µm] | 25 | 0.4 | 10 | Methanol : Phosphate buffer (22:78, v/v) | UV: 224 and 343 nm | 2.8-14 |
| **AMT** |  | 30 |  |  | Methanol : 0.1 % Formic Acid  (15:85, v/v) | QDa: 263 m/z | 0.2-4 |

API: Active Pharmaceutical Ingredient. CQ: Chloroquine phosphate. AMT: Artemether. LMF: Lumefantrine. T: Temperature. Q: Flow. Inj. Vol.: Injection volume.
