# Supplementary material for: Quality assessment of oral antimalarial and antiretroviral medicines used by public health systems in Sahel countries
Source: PLoS One. 2024 May 9;19(5):e0303289. doi: 10.1371/journal.pone.0303289 (PMC11081281; doi:10.1371/journal.pone.0303289)
Supplement: S2 Table — API: Active pharmaceutical ingredient. LPV: Lopinavir. RTV: Ritonavir. ABC: Abacavir sulphate. 3TC: Lamivudine. ZDV: Zidovudine. T: Temperature. Q: Flow. Inj. Vol.: Injection volume. *It is specified in the text. (DOCX) [file pone.0303289.s002.docx]

**S1 Table. Antiretroviral analytical method conditions.**

| **API** | **Column** | **T (ºC)** | **Q (ml/min)** | **Inj vol**  **(µL)** | **Mobile phase** | **Detection** | **Concentration Range (µg/ml)** |
| --- | --- | --- | --- | --- | --- | --- | --- |
| **LPV** | Acquity^TM^ Premier BEH C18 [100x2.1 mm, 1.7 µm] | 25 | 0.4 | 10 | ACN (55%)/ phosphate buffer (${KH}_{2}{PO}_{4}$, 45%), pH 4.0 | UV: 215 nm | 2.5 – 25 |
| **RTV** |  |  |  |  |  |  | 2.5 – 10 |
| **ABC** |  | 30 | Gradient* |  | Solution A (A)  Methanol (B) | UV: 270 nm | 3 – 18 |
| **3TC** |  |  |  |  |  |  | 1.5 – 9 |
| **ZDV** |  |  |  |  |  |  | 3 – 18 |

API: Active pharmaceutical ingredient. LPV: Lopinavir. RTV: Ritonavir. ABC: Abacavir sulphate. 3TC: Lamivudine. ZDV: Zidovudine. T: Temperature. Q: Flow. Inj. Vol.: Injection volume. *It is specified in the text.
