# Supplementary material for: Quality assessment of oral antimalarial and antiretroviral medicines used by public health systems in Sahel countries
Source: PLoS One. 2024 May 9;19(5):e0303289. doi: 10.1371/journal.pone.0303289 (PMC11081281; doi:10.1371/journal.pone.0303289)
Supplement: S3 Table — Data provided per unit (n) is expressed as CQ % released at each time. (DOCX) [file pone.0303289.s003.docx]

**S3 Table: Dissolution profile data of generic chloroquine phosphate medicine.**

| **n** | **Time (minutes)** | | | | | |
| --- | --- | --- | --- | --- | --- | --- |
|  | **7.5** | **15** | **22.5** | **30** | **37.5** | **45** |
| **1** | 0.00 | 45.24 | 75.56 | 85.39 | 83.68 | 86.76 |
| **2** | 0.00 | 28.11 | 64.63 | 83.59 | 86.08 | 86.75 |
| **3** | 0.00 | 41.17 | 71.57 | 82.51 | 92.34 | 91.02 |
| **4** | 0.00 | 7.85 | 49.59 | 66.59 | 77.99 | 84.20 |
| **5** | 0.00 | 39.09 | 69.52 | 81.12 | 87.67 | 86.10 |
| **6** | 0.00 | 5.01 | 71.58 | 83.86 | 84.57 | 83.86 |
| **7** | 0.00 | 0.00 | 48.53 | 74.93 | 81.32 | 77.85 |
| **8** | 19.13 | 57.32 | 85.03 | 86.55 | 86.49 | 88.60 |
| **9** | 24.59 | 66.98 | 85.70 | 86.16 | 87.14 | 90.13 |
| **10** | 0.00 | 36.46 | 70.78 | 83.47 | 88.25 | 89.26 |
| **11** | 0.00 | 51.63 | 79.70 | 88.26 | 84.50 | 86.37 |
| **12** | 0.00 | 42.47 | 76.36 | 83.82 | 88.48 | 91.74 |
| ***Mean*** | 3.64 | 35.11 | 70.71 | 82.19 | 85.71 | 86.89 |
| ***SD*** | 8.59 | 21.16 | 11.85 | 5.94 | 3.70 | 3.80 |
| ***CV*** | 235.73 | 60.26 | 16.76 | 7.23 | 4.31 | 4.37 |

Data provided per unit (n) is expressed as CQ % released at each time.
