# Supplementary material for: Quality assessment of oral antimalarial and antiretroviral medicines used by public health systems in Sahel countries
Source: PLoS One. 2024 May 9;19(5):e0303289. doi: 10.1371/journal.pone.0303289 (PMC11081281; doi:10.1371/journal.pone.0303289)
Supplement: S4 Table — Data provided per unit (n) is expressed as CQ % released at each time. (DOCX) [file pone.0303289.s004.docx]

**S4 Table: Dissolution profile data of Resochín® medicine.**

| **n** | **Time (minutes)** | | | | | |
| --- | --- | --- | --- | --- | --- | --- |
|  | **7.5** | **15** | **22.5** | **30** | **37.5** | **45** |
| **1** | 67.11 | 87.24 | 93.72 | 93.19 | 93.95 | 92.62 |
| **2** | 84.44 | 88.77 | 89.43 | 88.67 | 91.39 | 91.63 |
| **3** | 81.59 | 85.49 | 88.08 | 92.72 | 90.08 | 89.24 |
| **4** | 75.10 | 87.27 | 90.07 | 88.39 | 90.57 | 90.32 |
| **5** | 74.51 | 88.83 | 92.00 | 91.68 | 91.11 | 91.18 |
| **6** | 72.91 | 89.19 | 88.21 | 91.65 | 91.49 | 93.60 |
| **7** | 81.35 | 89.92 | 88.39 | 87.99 | 90.94 | 91.70 |
| **8** | 83.03 | 86.21 | 88.41 | 90.33 | 89.39 | 91.96 |
| **9** | 80.27 | 89.60 | 91.11 | 91.33 | 92.63 | 92.06 |
| **10** | 77.41 | 89.68 | 87.97 | 90.01 | 91.56 | 92.13 |
| **11** | 73.91 | 85.89 | 85.47 | 89.85 | 88.95 | 89.24 |
| **12** | 72.47 | 88.06 | 89.62 | 89.37 | 89.26 | 88.99 |
| ***Mean*** | 77.01 | 88.01 | 89.37 | 90.43 | 90.94 | 91.22 |
| ***SD*** | 5.19 | 1.56 | 2.16 | 1.69 | 1.44 | 1.47 |
| ***CV*** | 6.75 | 1.78 | 2.42 | 1.87 | 1.59 | 1.61 |

Data provided per unit (n) is expressed as CQ % released at each time.
